# Impact of Racist Microaggressions and LGBTQ-Related Minority Stressors: Effects on Psychological Distress Among LGBTQ+ Young People of Color

**DOI:** 10.5888/pcd20.220371

**Published:** 2023-07-20

**Authors:** John P. Salerno, M. V. Pease, Karina A. Gattamorta, Craig S. Fryer, Jessica N. Fish

**Affiliations:** 1 School of Social Work, Columbia University, New York, New York; 2 Department of Psychology, College of Behavioral and Social Sciences, University of Maryland, College Park, Maryland; 3 School of Nursing and Health Studies, University of Miami, Coral Gables, Florida; 4 Department of Behavioral and Community Health, School of Public Health, University of Maryland, College Park, Maryland; 5 Department of Family Science, School of Public Health, University of Maryland, College Park, Maryland

## Abstract

**Background:**

College students situated at the nexus of racial and sexual and gender minority (SGM) identities may experience multiple identity-related oppressions. We assessed whether racist microaggressions and lesbian, gay, bisexual, transgender, queer, or questioning (LGBTQ)–related minority stressors (ie, family rejection, identity concealment, racialized heterosexism and/or cisgenderism, internalized LGBTQ-phobia, and victimization) are associated with greater psychological distress among SGM college students of color (SOC) (students who identified as Hispanic/Latinx and/or any nonwhite race).

**Methods:**

Participants were a subset of SOC (n = 200) from a larger nonprobability cross-sectional study of SGM college students. Participants were recruited by using online social media platforms and university email listserves from May through August 2020. Participants completed an online Qualtrics survey using previously validated measures of minority stress, racist microaggressions, and psychological distress. Simple and covariate-adjusted multiple linear regression models were used to examine the associations between racist microaggressions and LGBTQ-related minority stressors with psychological distress.

**Results:**

In simple linear regression models, racist microaggressions and all LGBTQ-related stressors (ie, family rejection, identity concealment, racialized heterosexism and/or cisgenderism, internalized LGBTQ-phobia, and victimization) were significantly and positively associated with greater psychological distress. In covariate-adjusted multiple linear regression, racist microaggressions, internalized LGBTQ-phobia, and LGBTQ-related family rejection (but not identity concealment, racialized heterosexism and/or cisgenderism, and victimization) were independently and significantly associated with greater psychological distress.

**Conclusion:**

Study findings reveal that racist microaggressions, along with LGBTQ-related family rejection and internalized LGBTQ-phobia, have a significant impact on psychological distress among SGM SOC. Public health leaders have an important opportunity for policy and program development and reform to address the identity-related mental health needs of SGM SOC.

**Summary**

**What is already known on this topic?**

Racism and sexual and gender minority (SGM)–related stressors (eg, identity concealment, family rejection) have a detrimental effect on mental health among SGM young people of color (POC) and students of color (SOC) (those who identify as Hispanic/Latinx and/or any nonwhite race). Yet, how racist microaggressions affect SGM POC and SOC are seldom investigated.

**What is added by this report?**

Racist microaggressions perpetrated by white SGM people, along with SGM-related family rejection and internalized LGBTQ-phobia, have significant detrimental effects on psychological distress rates among SGM SOC.

**What are the implications for public health practice?**

Public health policy and program development and reform can address the identity-related mental health inequities faced by SGM SOC via implementation of antiracist, minority stress, and intersectionality-informed praxis.

## Introduction

The negative mental health impacts of interpersonal racism have been established (eg, racist discrimination, racist microaggressions) (1,2) and lesbian, gay, bisexual, transgender, queer, or questioning (LGBTQ)–related identity concealment (3), victimization (4), internalized homophobia (5), and family rejection (6) among sexual and gender minority (SGM) people and people of color (POC, anyone who identifies as Hispanic/Latinx and/or a nonwhite race). Further, existing research indicates that dual experiences of interpersonal racism and LGBTQ-related stressors (ie, gay rejection sensitivity, homonegativism, and heterosexism) have strong negative effects on the psychological health of SGM POC, including declines in mental well-being and increases in psychological distress, depression, and anxiety (1,7,8). However, few studies have examined the effects of interpersonal racism (ie, racist microaggressions) along with multiple forms of LGBTQ-related stress (ie, identity concealment, family rejection, internalized homophobia, victimization). Given existing literature linking more singular forms of LGBTQ-related stress and interpersonal racism with their negative effects on mental health, this is a substantial gap in the scientific literature. Unfortunately, the experience of LGBTQ-related stressors may be complicated when considering interpersonal racism (stressors that are unique to POC), including anti-Black (9) and anti-Asian (10) racism or racism-related stressors, as well as anti-immigrant and anti-Latinx discrimination and xenophobia (11). Additionally, interpersonal racism against SGM POC can often manifest as an intracommunity form of discrimination, in which SGM POC are racially discriminated against by (often white) SGM people (12,13). Given that 41% of all young persons in the US are currently enrolled in college, nearly half (45%) of which are POC (14), and the identity-related vulnerabilities faced by SGM POC, it is highly important to investigate the unique effects of multiple identity-related stressors that are likely affecting the mental health of SGM students of color (SOC, any college student who identifies as Hispanic/Latinx and/or a nonwhite race), such as interpersonal racism and LGBTQ-related minority stressors. The risk for the multiple experience of LGBTQ-related minority stressors and interpersonal racism may place the mental well-being of SGM SOC in a hypervulnerable position.

Our study used the minority stress theory (15,16), which emphasizes the role of externalized (eg, discriminatory events perpetrated against SGM persons) and internalized (eg, homophobic feelings related to SGM identities) minority stressors and their effects on mental health among SGM persons. Our study implemented minority stress theory by including measures of LGBTQ-related identity concealment, family rejection, victimization, internalized LGBTQ-phobia, and racialized heterosexism and/or cisgenderism. This study also used Critical Race Theory (CRT) (17,18), a methodologic framework focused on countering racist systems of oppression that lead to health inequities that negatively affect POC. Specifically, our study employs CRT by 1) acknowledging that racism is a central social determinant of health inequities among POC, 2) focusing on groups of people who are disadvantaged and marginalized by their social positionalities and aiming to amplify their experiences and needs (ie, SGM SOC), 3) highlighting racism as a substantial and relevant contributor to the mental health outcome of interest (ie, racist microaggressions and LGBTQ-related stressors affect psychological distress), and 4) acknowledging that racism is a structural issue that manifests interpersonally through common, every day, and frequent occurrences, such as racist microaggressions, which are substantial and impactful forms of racism. Our study was further guided by the intersectionality principle of CRT, which emphasizes that the amalgamation of vulnerable social positionalities and multiple forms of oppression likely produce mental health–related inequities (17,18); in our study, we used intersectionality to understand multiple forms of oppression (ie, LGBTQ-related minority stressors and racist microaggressions) among SGM SOC and how they relate to psychological distress.

We examined whether racist microaggressions, along with multiple LGBTQ-related minority stressors (ie, identity concealment, family rejection, victimization, internalized LGBTQ-phobia, and racialized heterosexism and/or cisgenderism), are associated with greater psychological distress. We hypothesized that racist microaggressions and all LGBTQ-related minority stressors would significantly and positively associate with greater psychological distress. The results of this study may provide useful information relevant for developing, reforming, and changing policies, practices, and programs to address racial, sexual, and gender minority identity–related oppression and mental health needs among SGM SOC.

## Methods

### Study design and sample

The data analyzed in this study were derived from nonprobability cross-sectional data collected from a sample of SGM students (N = 565) to explore minority stress and mental health among SGM college students. A subset of racial and ethnic minority students (n = 200) was used for this analysis (identified as Hispanic/Latinx or a nonwhite race or both). Eligibility criteria for the parent study included being at least 18 years of age, identifying as a sexual or gender minority person, and being a full-time, currently enrolled college student. Data were collected from May 27 through August 14, 2020, via online survey. Participants were incentivized with a raffle for a $50 Amazon gift card. Participants were recruited by using an electronic recruitment flyer distributed via multiple social media platforms that included a link to an online Qualtrics survey. Additionally, recruitment occurred through email campaigns within the study implementation team’s internal and external professional networks, and at historically Black colleges and universities (HBCUs), Hispanic-serving institutions, and LGBTQ+ student centers across the US. University of Maryland institutional review board approval and participant informed consent were obtained before commencing data collection. Survey duration was approximately 20 to 25 minutes. On completion of the survey, participants were provided a list of mental health and crisis management resources and contact information for the study principal investigator.

### Measures

#### Racist microaggressions (perpetrated by white LGBTQ people)

Racist microaggressions were measured by using an adapted version of 5 items from the LGBT People of Color Microaggressions Scale (19). To capture past year racist microaggressions, students were asked to indicate whether they experienced each item in the past year (yes/no). The scale demonstrated strong internal consistency in the current sample (α = .85). A composite score was calculated by summing responses across the 5 items (range, 0–5; mean [SD], 1.90 [2.01]).

#### LGBTQ-related identity concealment

LGBTQ-related identity concealment was measured by using an adapted version of 3 items from the LGBT Minority Stress Measure (LMSM) (20) and 4 items from the Daily Heterosexist Experiences Questionnaire (DHEQ) (21). To capture past year identity concealment, students were asked to indicate whether they experienced each item in the past year (yes/no). A composite score was calculated for all 7 items (range, 0–7; mean [SD], 3.76 [2.09]). The scale demonstrated strong internal consistency in the current sample (α = .79).

#### LGBTQ-related victimization

Victimization was measured by using an adapted version of 4 items from the DHEQ (21) and 2 items from the LMSM (20). To capture presence of past year victimization, students were asked to indicate whether they experienced each item in the past year (yes/no). A composite score was calculated by summing responses across the 6 items (range, 0–6; mean [SD], 0.41 [0.89]; α = .66).

#### LGBTQ-related family rejection

Family rejection was measured by using an adapted version of 10 items from the Sexual Minority Adolescent Stress Inventory (22) and 7 items from the DHEQ (21). To capture past year family rejection, students were asked to indicate whether they experienced each item in the past year (yes/no). A composite score was calculated by summing responses across the 17 items (range, 0–17; mean [SD], 5.89 [4.14]). The scale demonstrated strong internal consistency in our sample (α = .86).

#### Internalized LGBTQ-phobia

Internalized LGBTQ-phobia was measured by using an adapted version of 7 items from the LMSM (20). To capture past year internalized LGBTQ-phobia, students were asked to indicate whether they experienced each item in the past year (yes/no). A composite score was calculated by summing responses across the 7 items (range, 0–7; mean [SD], 1.06 [1.89]). The scale demonstrated strong internal consistency in our sample (α = .83).

#### Racialized heterosexism and/or cisgenderism

Racialized heterosexism and/or cisgenderism was measured by using an adapted version of 5 items from the LGBT People of Color Microaggressions Scale (19). To capture past year racialized heterosexism and/or cisgenderism, students were asked to indicate whether they experienced each item in the past year (yes/no). A composite score was calculated by summing responses across the items (range, 0–5; mean [SD], 1.21 [1.53]; α = .63).

#### Psychological distress

The previously validated Kessler-10 (23,24) was used to measure current psychological distress among SGM SOC. The 10-item scale includes measures of depression and anxiety in the past 30 days. Item responses are coded on a 5-point Likert-type scale from none of the time (1) to all of the time (5). A composite score was calculated for all 10 items (range, 0–40; mean [SD], 17.44 [8.36]). The measure showed strong internal consistency in our sample (α = .92).

#### Covariates

Linear regression models were adjusted for continuous social isolation (25), parental financial dependence, and age, and adjusted for dichotomous sex assigned at birth (1 = female, 0 = male), gender identity (1 = noncisgender identity, 0 = cisgender identity), sexual orientation (1 = any nonbisexual sexual orientation, 0 = bisexual orientation), educational program (1 = undergraduate, 0 = graduate), Latinx ethnicity (1 = Latinx, 0 = not Latinx), race (1 = any nonwhite race, 0 = white race), living with parents (1 = living with parents, 0 = not living with parents), and being out to parents (1 = out to parents, 0 = not out to parents).

### Analytic strategy

Data management and analyses were conducted by using IBM SPSS Statistics for Mac, Version 28 (IBM). First, descriptive statistics of sociodemographic characteristics and their associations with psychological distress (using independent samples *t* tests and 1-way analysis of variance [ANOVA] *F* tests) were conducted. Afterward, the bivariate associations between past year LGBTQ-related minority stressors, and racist microaggressions, with psychological distress were examined by using simple linear regression. Next, covariate-adjusted multiple linear regression modeling was used to examine the associations between past year LGBTQ-related minority stressors, and racist microaggressions, with psychological distress; interactions between significant LGBTQ-related stressors and racist microaggressions were examined. Alpha was set to less than .05.

## Results

### Sociodemographic characteristics and psychological distress

Among SGM SOC in the sample, more than half identified as female (72%) and cisgender (gender identity that corresponds with sex assigned at birth; 71%); both sex assigned at birth (*P* < .001) and gender identity (*P* = .02) were significantly associated with psychological distress (Table 1). Although sexual orientation varied, bisexuality (30%) was the most prevalent; sexual orientation (*P* = .02) was significantly associated with psychological distress. Most of the sample were undergraduate (74%) students, and average age was 21.7 years; age and educational program were not significantly associated with psychological distress. A total of 38% of sample participants identified their ethnicity as Hispanic or Latinx. A plurality identified their race as white (39%), Asian (36%), Black or African American (26%), and/or another nonwhite race (19%; including Native Hawaiian or Pacific Islander, American Indian or Alaska Native, and a race not listed); only white race (*P* = .01) was significantly associated with psychological distress. Most of the sample indicated that they were financially dependent on their parents at some level (85%); parental financial dependence was not significantly associated with psychological distress. Approximately 64% were out to their parents and currently living with them; being out to parents and living with parents were not significantly associated with psychological distress.

**Table 1:** Sample Sociodemographic Characteristics Among Sexual and Gender Minority College Students of Color^a^ (N = 200) and Their Associations with Psychological Distress

Variables	N (%)^b^	Psychological distress, mean (SD)	*P* value
**Sex assigned at birth **			
Male	55 (28)	14.2 (8.4)	.001
Female	143 (72)	18.5 (8.0)	
**Gender identity^c^**			
Cisgender	141 (71)	16.3 (8.4)	.02
Transgender	13 (7)	23.2 (7.0)	
Nonbinary	25 (13)	20.8 (8.4)	
Genderqueer	11 (6)	18.4 (6.8)	
Two-spirit	2 (1)	17.5 (6.4)	
Gender fluid	3 (2)	11.3 (10.2)	
Agender	5 (3)	24.4 (4.4)	
**Sexual orientation^c^**			
Bisexual	59 (30)	17.6 (8.3)	.02
Gay	37 (19)	13.1 (8.3)	
Lesbian	31 (16)	17.9 (8.7)	
Queer	33 (17)	20.8 (7.7)	
Same gender loving	4 (2)	18.2 (8.1)	
Pansexual	15 (8)	26.4 (7.2)	
Questioning	3 (2)	17.7 (3.8)	
Nonbinary	1 (1)	33.0 (0)	
Heterosexual/straight	1 (1)	24.0 (0)	
Asexual	16 (8)	18.5 (8.0)	
**Age, mean (SD), y**	21.7 (3.79)	NA	.53
**Hispanic or Latinx**			
Yes	75 (38)	18.1 (8.2)	.40
No	125 (63)	17.1 (8.5)	
**Race^d^**			
American Indian/Alaska Native	8 (4)	17.8 (10.3)	.91
Asian	72 (36)	16.2 (7.8)	.13
Black or African American	52 (26)	17.7 (8.6)	.77
Native Hawaiian or Pacific Islander	7 (4)	21.6 (8.2)	.18
White	77 (39)	19.3 (8.2)	.01
Another race not listed^e^	22 (11)	21.6 (8.8)	.01
**Living with parents**			
Yes	127 (64)	16.7 (8.0)	.11
No	73 (37)	18.7 (8.9)	
**Parental financial dependence, mean (SD)**	3.4 (1.4)	NA	.25
**Out to parents**			
Yes	128 (64)	17.1 (8.3)	.39
No	71 (36)	18.2 (8.6)	
**Educational program**			
Undergraduate	148 (74)	17.5 (8.4)	.79
Graduate	52 (26)	17.2 (8.4)	
**Social isolation^f^, mean (SD)**	6.45 (1.74)	NA	<.001

Abbreviation: NA, not applicable.

a Students who identified as Hispanic/Latinx and/or any nonwhite race. The authors have chosen to not capitalize “white” to stand in solidarity with the antiracism movement.

b Data are n (%) unless otherwise noted. Numbers may not add to 200 because of missing data.

c Gender identity and sexual orientation were collected by asking participants to select the identity that best represented their sexual orientation and gender identity; all potential options are listed here.

d Total percent in this category will not add up to 100, as participants were instructed to select all that apply; all potential race options are listed here.

e Those who selected another race not listed specified Latin American, Arab, and Middle Eastern native identities, as well as mixed race, biracial, and Indigenous identities.

f The social isolation scores range from 3 to 9, with higher scores indicating more social isolation (25).

### Bivariate associations with psychological distress

Bivariate relationships (ie, simple linear regression) between LGBTQ-related stressors, and racist microaggressions, with psychological distress are reported in Table 2. Past year racist microaggressions (*P* < .001), and LGBTQ-related identity concealment (*P* = .049), internalized LGBTQ-phobia (*P* < .001), family rejection (*P* < .001), victimization (*P* = .01), and racialized heterosexism and/or cisgenderism (*P* = .002) were all significantly and positively associated with greater psychological distress.

**Table 2:** Racist Microaggressions and LGBTQ-Related Minority Stressor Scores and Associations with Psychological Distress Among Sexual and Gender Minority College Students of Color^a^ (N = 200)

Subscales and items^b^	Mean (SD)	*t* value^c^	*P* value^c^
**Past year racist microaggressions (range, 0–5)**	1.90 (2.01)	4.38	<.001
1. Not being able to trust white LGBTQ people			
2. Feeling misunderstood by white LGBTQ people			
3. Having to educate white LGBTQ people about race issues			
4. Being told that “race isn’t important” by white LGBTQ people			
5. White LGBTQ people saying things that are racist			
**Past year identity concealment (range, 0–7)**	3.76 (2.09)	1.98	.049
1. Avoided telling people about certain things in my life that might imply I am LGBTQ			
2. Avoided talking about my romantic life because I do not want others to know I am LGBTQ			
3. Did not object when I heard anti-LGBTQ remarks because I did not want others to assume I am LGBTQ			
4. Watched what I said and did around heterosexual people			
5. Pretended that I had an opposite-sex partner			
6. Pretended that I was heterosexual			
7. Hid part of my life from other people			
**Past year internalized LGBTQ-phobia (range, 0–7)**	1.06 (1.89)	4.80	<.001
1. If I was offered the chance to be someone who is not LGBTQ, I would accept the opportunity			
2. I wish I wasn’t LGBTQ			
3. I envy people who are not LGBTQ			
4. I feel that being LGBTQ is a personal flaw in me			
5. I feel that me being LGBTQ must have been a mistake of fate or nature			
6. I wonder why I am not “normal” and like everyone else			
7. I have tried to stop being LGBTQ			
**Past year family rejection (range, 0–17)**	5.89 (4.14)	4.38	<.001
1. Being rejected by my mother for being LGBTQ			
2. Being rejected by my father for being LGBTQ			
3. Being rejected by my legal guardian for being LGBTQ			
4. Being rejected by a sibling or siblings because I am LGBTQ			
5. Being rejected by other relatives because I am LGBTQ			
6. Family members not accepting your partner as part of the family			
7. Family members avoiding talking about your LGBTQ identity			
8. Hearing family members make negative comments about LGBTQ people			
9. Lying to my family about being LGBTQ			
10. If I come out, it will cause problems in my family			
11. My family does not want to talk to me about being LGBTQ			
12. Someone who lives with me has told me they disapprove of me being LGBTQ			
13. I feel as though I am a disappointment to my family because I am LGBTQ			
14. My family has told me that being LGBTQ is just a phase			
15. My parents are uncomfortable with LGBTQ people			
16. My parents are sad that I am LGBTQ			
17. My family tries to make me straight			
**Past year victimization (range, 0–6)**	0.41 (0.89)	2.48	.01
1. Being threatened with harm because I am LGBTQ			
2. Being bullied by others because I am LGBTQ			
3. Being punched, kicked, or beaten because I am LGBTQ			
4. Being assaulted with a weapon because I am LGBTQ			
5. Being raped or sexually assaulted because I am LGBTQ			
6. Having objects thrown at me because I am LGBTQ			
**Past year racialized heterosexism and/or cisgenderism (range, 0–5)**	1.21 (1.53)	3.16	.002
1. Not being accepted by other people of your race/ethnicity because you are LGBTQ			
2. Feeling misunderstood by people in your ethnic/racial community			
3. Difficulty finding friends who are LGBTQ and from your racial/ethnic background			
4. Feeling unwelcome at groups or events in your racial/ethnic community			
5. Not having LGBTQ people of color as positive role models			

Abbreviation: LGBTQ, lesbian, gay, bisexual, transgender, queer, or questioning.

a Students who identified as Hispanic/Latinx and/or any nonwhite race. The authors have chosen to not capitalize “white” to stand in solidarity with the antiracism movement.

b Base question for all constructs: “Has this happened to you in the past year?”; item response options: 1 = yes, 0 = no.

c Simple linear regression was used to examine associations between LGBTQ-related minority stress and race-related variables with psychological distress.

### Multivariable associations with psychological distress

Covariate-adjusted multiple linear regression modeling that tested the associations of past year racist microaggressions, and LGBTQ-related stressors, with psychological distress are reported in Table 3. The model showed that, independently, racist microaggressions (*B* **=** 0.75 [95% CI, 0.15–1.35]; β = 0.18; *P* = .02), family rejection (*B* **=** 0.30 [95% CI, 0.01–0.59]; β = 0.15; *P* = .04), and internalized LGBTQ-phobia (*B* **=** 0.93 [95% CI, 0.33–1.54]; β = 0.21; *P* = .003) in the past year each were significantly and positively associated with greater psychological distress. LGBTQ-related identity concealment, victimization, and racialized heterosexism and/or cisgenderism were no longer significantly associated with greater psychological distress. Additionally, social isolation (*B* **=** 0.95 [95% CI, 0.33–1.57]; β = 0.20; *P* = .003), another race not listed (*B* **=** 3.14 [95% CI, 0.55–5.74]; β = 0.15; *P* = .02), and white race (*B* **=** 3.09 [95% CI, 1.01–5.17]; β = 0.18; *P* = .004) were significantly and positively associated with greater psychological distress. All other covariates were not significantly associated with greater psychological distress.

**Table 3:** Covariate-Adjusted Multiple Linear Regression Model Testing the Associations of Past Year Racist Microaggressions and LGBTQ-Related Minority Stressors with Psychological Distress Among Sexual and Gender Minority College Students of Color^a^ (N = 200)

Independent variables	*B* (95% CI)	β	*P* value	Effect size^b^
**Racist microaggressions (continuous)**	0.75 (0.15 to 1.35)	0.18	.02	0.19
**Family rejection (continuous)**	0.30 (0.01 to 0.59)	0.15	.04	0.15
**Victimization (continuous)**	0.13 (−1.11 to 1.37)	0.01	.84	0.02
**Identity concealment (continuous)**	0.17 (−0.35 to 0.69)	0.04	.52	0.05
**Racialized heterosexism and/or cisgenderism (continuous)**	−0.12 (−0.91 to 0.67)	−0.02	.76	−0.02
**Internalized LGBTQ-phobia (continuous)**	0.93 (0.33 to 1.54)	0.21	.003	0.23
**Sexual orientation**				
Gay	−1.87 (−5.72 to 1.97)	−0.09	.34	−0.07
Lesbian	0.75 (−2.40 to 3.89)	0.03	.64	0.04
Queer	0.47 (−2.72 to 3.66)	0.02	.77	0.02
Another sexual orientation	−0.36 (−3.32 to 2.60)	−0.02	.81	−0.02
Bisexual	1 [Reference]			
**Race**				
**Another race not listed**	3.14 (0.55 to 5.74)	0.15	.02	0.18
All who identified as something else	1 [Reference]			
**White**	3.09 (1.01 to 5.17)	0.18	.004	0.22
All who identified as something else	1 [Reference]			
**Gender identity**				
Noncisgender	2.10 (−0.25 to 4.45)	0.11	.08	0.13
Cisgender	1 [Reference]			
**Social isolation (continuous)**	0.95 (0.33 to 1.57)	0.20	.003	0.23
**Sex assigned at birth**				
Female assigned at birth	3.20 (−0.58 to 6.46)	0.17	.05	0.15
Male assigned at birth	1 [Reference]			

Abbreviation: LGBTQ, lesbian, gay, bisexual, transgender, queer, or questioning.

a Students who identified as Hispanic/Latinx and/or any nonwhite race. The authors have chosen to not capitalize “white” to stand in solidarity with the antiracism movement.

b Partial η^2^.

Post hoc analyses testing the interaction of past year racist microaggressions and family rejection (*B* = −0.01 [95% CI, −0.14 to 0.12]; β = 0.65; *P* = .85), and past year racist microaggressions and internalized LGBTQ-phobia (*B* **=** −0.23 [95% CI, −0.53 to 0.06]; β = −0.17; *P* = .11) demonstrated that these interactions were not significantly associated with greater psychological distress.

## Discussion

Our study aimed to explore the effect of past year racist microaggressions, and LGBTQ-related minority stressors, and their association with psychological distress among SGM SOC. Our study hypothesis was partially supported: racist microaggressions and LGBTQ-related family rejection and internalized LGBTQ-phobia were associated with greater psychological distress. Though LGBTQ-related identity concealment, victimization, and racialized heterosexism and/or cisgenderism were significant in initial bivariate models, they were no longer significant in the final multivariable model. Study findings indicate that racist microaggressions, along with LGBTQ-related family rejection and internalized LGBTQ-phobia, may have a unique effect on the mental well-being of SGM SOC; the findings highlight the importance of using minority stress theory, CRT, and intersectionality framing in tandem toward eliminating the mental health inequities faced by SGM SOC.

In multivariable analyses, racist microaggressions, LGBTQ-related family rejection, and internalized LGBTQ-phobia were associated with greater psychological distress. These findings contribute to previous research, which has documented significant associations of racism and other LGBTQ-related stressors (ie, heterosexism, gay rejection sensitivity, and homonegativism) on mental health among SGM POC (1,2,7,8). These results add to existing research by further documenting that racist microaggressions and multiple forms of LGBTQ-related minority stressors are impactful predictors for psychological distress among SGM SOC. More research and interventions are needed to understand and address the intersectional identity-related mental health needs of these populations. Study findings are concerning given that SGM SOC are still emerging in their adulthood. Developmentally, they are in a vulnerable stage of the life course during which they are working toward establishing themselves as independent adults. Though all college students face this particular vulnerability and threat to their mental health, SGM SOC uniquely face identity-related (ie, LGBTQ+, race, and ethnicity) mental health inequities (ie, racist microaggressions, internalized LGBTQ-phobia, and family rejection), as revealed by our study findings. These unique identity-related stressors can hinder the developmental process in addition to affecting mental health. Therefore, these stressors may have long-term implications for students’ health and well-being across the lifespan.

The fact that racist microaggressions remained a significant predictor in the final model, while some LGBTQ-related minority stressors did not, is noteworthy. Much of the existing minority stress research literature is not inclusive of racial identity–related stressors and their impact on mental health; our study findings suggest that racist microaggressions are at least as impactful toward the mental health of SGM SOC compared with LGBTQ-related minority stressors. Lastly, study findings on racist microaggressions are meaningful, in that they highlight 1) the power imbalance that exists between white SGM people and SGM POC (students in this case), 2) the manifestation of this power imbalance as racist microaggressions perpetrated by white SGM people, and 3) the consequential mental health inequity faced by SGM SOC (ie, greater psychological distress). These intracommunity racist microaggressions likely have implications for LGBTQ+ community belonging among SGM SOC and POC and warrant further investigation into intracommunity stress faced by SGM SOC and POC and the effect of this stress on their mental health and well-being. There is a great need to move beyond traditional models of minority stress toward combination frameworks that can reduce the unique identity-related inequities faced by SGM SOC and POC (eg, CRT, intersectionality, life-course perspective, minority stress theory).

Public health, mental health, and higher education responses and practices rooted in intersectional and antiracist frameworks are necessary and must address stressors above and beyond those singularly related to one’s sexual orientation, gender identity, and race and ethnicity. There is a great need for culturally sensitive (toward racial and LGBTQ+ minority young people and students) and antiracist practice and response efforts to address racism and non–race-related SGM stressors (eg, LGBTQ-related family rejection) among SGM SOC. Study results overall suggest that racist microaggressions and some LGBTQ-related stressors (ie, family rejection and internalized LGBTQ-phobia) have a unique impact on the mental health of SGM SOC.

### Limitations

Although our study has many strengths, there are limitations to consider. For instance, this study used a nonprobability and cross-sectional sampling strategy, which limits our ability to generalize findings to broader populations and to make causal assessments. Further, a small overall sample size, as well as small cell sizes, likely affected statistical power and ability to detect significant interactions between LGBTQ-related minority stressors and racist microaggressions. The study also implemented a retrospective self-report data collection strategy, which increases risk for recall and social desirability biases. Additionally, our study measure of racist microaggressions only captured these microaggressions perpetrated by white LGBTQ people, which likely underestimates the overall experience of racist microaggressions perpetrated by white people in general. However, significant findings were still detected, suggesting the salient impact of measured and unmeasured forms of racist microaggressions. Further, linear models and quantitative data have the potential to misrepresent the realities of SGM POC because of limitations in being able to capture the complexity of sociohistorical-demographic diversity within and between groups. Future researchers should intentionally center the needs of SGM SOC to allow for analyses examining within-group differences across diverse categories of sex, gender identity, sexual orientation, age, race, ethnicity, and nationality, and those marginalized by precarious socioeconomic positions. Lastly, the study was conducted during the first months of the COVID-19 pandemic, which may have had an influence on study findings. Yet, the study reveals how LGBTQ-related stressors and racist microaggressions affect mental health among SGM SOC amid the COVID-19 pandemic, and highlights the importance of intervention, practice, and policy to address the intersectionality of mental health challenges among SGM SOC and POC.

### Public health implications

#### Practice implications for serving SGM SOC

Study findings have public health implications relevant to mental health among SGM SOC. First, our findings indicate that racist microaggressions in the past year are associated with greater rates of psychological distress, including when adjusting for all LGBTQ-related stressors, highlighting that SGM SOC are uniquely affected by racist microaggressions above and beyond LGBTQ-related stressors alone. Study results also indicated that SGM SOC are uniquely affected by LGBTQ-related internalized LGBTQ-phobia and family rejection. Therefore, university and off-campus therapists, and online, text, and hotline crisis, mental health, and social support resources should intentionally prioritize extending access to culturally sensitive mental health and social support services for SGM SOC by increasing visible allyship (eg, brown and black stripes on pride rainbows), increasing financial accessibility of services, representation of SGM providers of color, and providers’ awareness and competency in addressing intersectional mental health concerns. The public health, mental health, and higher education (and K-12) communities are urged to act by supporting the hiring of professionals with training and expertise in LGBTQ+, antiracism, intersectionality, and social justice concerns as well as the dissemination of existing minority stress reduction (26,27) and family acceptance (28) resources within mental health and education contexts. Professionals who can center the urgent need for antiracist and intersectional framing of these resources (29–31) that addresses racism as well as LGBTQ-related stressors experienced by SGM SOC are desperately needed. Lastly, higher education (and K-12) communities (eg, LGBTQ+ student center directors, administrative leaders, professors, teachers, counselors, principals), particularly at predominantly White institutions (PWI), given the greater risk for white-centered LGBTQ equity efforts and racism perpetrated by white LGBTQ people, are implored to engage in antiracist and intersectional inclusive response practices and advocacy (32–34) that 1) fully embrace SGM SOC and those with other marginalized statuses and identities (eg, undocumented immigrants, foreign nationals, and non-Christian religion affiliated), 2) aim to eliminate mental health inequity driven by racism and other identity-related oppressions (eg, LGBTQ-related minority stress), and 3) promote increases in opportunities that well position SGM SOC to thrive and succeed across the life course. Useful models of relevant programs and initiatives can be found in work done at HBCUs, where efforts to provide critical intersectional and inclusive support for SGM SOC are ongoing, including developing inclusive admissions policies; providing affirmative and culturally cognizant health care, therapy, and peer education services; and establishing gender-inclusive housing programs (35,36).

#### Implications for future research

It is critical for future researchers to replicate and extend our findings and continue to investigate the role of racism in pathways of LGBTQ+ minority stress to mental health, which differ substantially from examining SGM stress among SGM POC, or considering general discrimination in studies of SGM stress. More research is needed to develop and psychometrically test tools that assess different types of racist experiences among SGM POC and are applicable to the experiences of subgroups within SGM POC (eg, by differences in sexual orientation, gender identity, race, ethnicity, nationality, and immigration status). Such research is necessary for effective mental health, public health, and higher education interventions that work toward the elimination of racism and other types of social and health injustices and inequities. As public health scholars, we have a responsibility to address health disparities and inequities that are maintained through social systems of marginalization and oppression, such as racism. Intentional health equity approaches are urgently needed in public health research to eliminate health disparities and inequities among historically marginalized groups, such as Black, Latinx, Indigenous, and other POC, as well as LGBTQ+ people. It is imperative to apply critical social paradigms, such as CRT, intersectionality, and minority stress theory, in combination to address inequality at the nexus of SGM and racial or ethnic minority identities or both. Such frameworks aim to eliminate health inequities by revealing and challenging complex structural and historical systems of power and oppression that continue to reinforce multiple forms of social exclusion to the detriment of the health and well-being of LGBTQ+ young POC.

## References

[1] Velez BL , Polihronakis CJ , Watson LB , Cox R Jr . Heterosexism, racism, and the mental health of sexual minority people of color. Couns Psychol 2019;47(1):129–59. 10.1177/0011000019828309

[2] Salerno JP , Gattamorta KA , Williams ND . Impact of family rejection and racism on sexual and gender minority stress among LGBTQ young people of color during COVID-19. Psychol Trauma 2022. 10.1037/tra0001254 35511543 PMC10361835

[3] Pachankis JE , Mahon CP , Jackson SD , Fetzner BK , Bränström R . Sexual orientation concealment and mental health: a conceptual and meta-analytic review. Psychol Bull 2020;146(10):831–71. 10.1037/bul0000271 32700941 PMC8011357

[4] Seelman K , Woodford MR , Nicolazzo Z . Victimization and microaggressions targeting LGBTQ college students: gender identity as a moderator of psychological distress. J Ethn Cult Divers Soc Work 2017;26(1-2):112–25. 10.1080/15313204.2016.1263816

[5] Newcomb ME , Mustanski B . Internalized homophobia and internalizing mental health problems: a meta-analytic review. Clin Psychol Rev 2010;30(8):1019–29. 10.1016/j.cpr.2010.07.003 20708315

[6] Mitrani VB , De Santis JP , McCabe BE , Deleon DA , Gattamorta KA , Leblanc NM . The impact of parental reaction to sexual orientation on depressive symptoms and sexual risk behavior among Hispanic men who have sex with men. Arch Psychiatr Nurs 2017;31(4):352–8. 10.1016/j.apnu.2017.04.004 28693870 PMC5721521

[7] English D , Rendina HJ , Parsons JT . The effects of intersecting stigma: a longitudinal examination of minority stress, mental health, and substance use among Black, Latino, and multiracial gay and bisexual men. Psychol Violence 2018;8(6):669–79. 10.1037/vio0000218 30881729 PMC6415673

[8] Layland EK , Maggs JL , Kipke MD , Bray BC . Intersecting racism and homonegativism among sexual minority men of color: latent class analysis of multidimensional stigma with subgroup differences in health and sociostructural burdens. Soc Sci Med 2022;293:114602. 10.1016/j.socscimed.2021.114602 34933242 PMC9020748

[9] Bor J , Venkataramani AS , Williams DR , Tsai AC . Police killings and their spillover effects on the mental health of Black Americans: a population-based, quasi-experimental study. Lancet 2018;392(10144):302–10. 10.1016/S0140-6736(18)31130-9 29937193 PMC6376989

[10] Tessler H , Choi M , Kao G . The anxiety of being Asian American: hate crimes and negative biases during the COVID-19 pandemic. Am J Crim Justice 2020;45(4):636–46. 10.1007/s12103-020-09541-5 32837158 PMC7286555

[11] Garcini LM , Domenech Rodríguez MM , Mercado A , Silva M , Cadenas G , Galvan T , . Anti-immigration policy and mental health: risk of distress and trauma among Deferred Action for Childhood Arrivals recipients in the United States. Psychol Trauma 2022. 10.1037/tra0001228 35482682 PMC9867934

[12] Jackson SD , Mohr JJ , Sarno EL , Kindahl AM , Jones IL . Intersectional experiences, stigma-related stress, and psychological health among Black LGBQ individuals. J Consult Clin Psychol 2020;88(5):416–28. 10.1037/ccp0000489 32091225

[13] Han CS , Ayala G , Paul JP , Boylan R , Gregorich SE , Choi KH . Stress and coping with racism and their role in sexual risk for HIV among African American, Asian/Pacific Islander, and Latino men who have sex with men. Arch Sex Behav 2015;44(2):411–20. 10.1007/s10508-014-0331-1 25060122 PMC4305487

[14] Espinosa LL , Turk JM , Taylor M , Chessman HM . Race and ethnicity in higher education: a status report. 2019. Accessed May 16, 2023. https://www.equityinhighered.org/wp-content/uploads/2019/02/Race-and-Ethnicity-in-Higher-Education.pdf

[15] Meyer IH . Prejudice, social stress, and mental health in lesbian, gay, and bisexual populations: conceptual issues and research evidence. Psychol Bull 2003;129(5):674–97. 10.1037/0033-2909.129.5.674 12956539 PMC2072932

[16] Brooks VR . Minority stress and lesbian women. Free Press; 1981.

[17] Ford CL , Airhihenbuwa CO . The public health critical race methodology: praxis for antiracism research. Soc Sci Med 2010;71(8):1390–8. 10.1016/j.socscimed.2010.07.030 20822840

[18] Solórzano DG , Yosso TJ . Critical race methodology: counter-storytelling as an analytical framework for education research. Qual Inq 2002;8(1):23–44. 10.1177/107780040200800103

[19] Balsam KF , Molina Y , Beadnell B , Simoni J , Walters K . Measuring multiple minority stress: the LGBT People of Color Microaggressions Scale. Cultur Divers Ethnic Minor Psychol 2011;17(2):163–74. 10.1037/a0023244 21604840 PMC4059824

[20] Outland PL . Developing the LGBT Minority Stress Measure [master’s thesis]. [Fort Collins (CO)]: Colorado State University; 2016.

[21] Balsam KF , Beadnell B , Molina Y . The Daily Heterosexist Experiences Questionnaire: measuring minority stress among lesbian, gay, bisexual, and transgender adults. Meas Eval Couns Dev 2013;46(1):3–25. 10.1177/0748175612449743 24058262 PMC3777637

[22] Schrager SM , Goldbach JT , Mamey MR . Development of the Sexual Minority Adolescent Stress Inventory. Front Psychol 2018;9:319. 10.3389/fpsyg.2018.00319 29599737 PMC5862853

[23] Kessler RC , Andrews G , Colpe LJ , Hiripi E , Mroczek DK , Normand SL , . Short screening scales to monitor population prevalences and trends in non-specific psychological distress. Psychol Med 2002;32(6):959–76. 10.1017/S0033291702006074 12214795

[24] Kessler RC , Barker PR , Colpe LJ , Epstein JF , Gfroerer JC , Hiripi E , . Screening for serious mental illness in the general population. Arch Gen Psychiatry 2003;60(2):184–9. 10.1001/archpsyc.60.2.184 12578436

[25] Hughes ME , Waite LJ , Hawkley LC , Cacioppo JT . A short scale for measuring loneliness in large surveys: results from two population-based studies. Res Aging 2004;26(6):655–72. 10.1177/0164027504268574 18504506 PMC2394670

[26] Pachankis JE , McConocha EM , Reynolds JS , Winston R , Adeyinka O , Harkness A , . Project ESTEEM protocol: a randomized controlled trial of an LGBTQ-affirmative treatment for young adult sexual minority men’s mental and sexual health. BMC Public Health 2019;19(1):1086. 10.1186/s12889-019-7346-4 31399071 PMC6688287

[27] Pachankis JE , McConocha EM , Clark KA , Wang K , Behari K , Fetzner BK , . A transdiagnostic minority stress intervention for gender diverse sexual minority women’s depression, anxiety, and unhealthy alcohol use: a randomized controlled trial. J Consult Clin Psychol 2020;88(7):613–30. 10.1037/ccp0000508 32437174 PMC7597069

[28] Diamond GM , Boruchovitz-Zamir R , Nir-Gotlieb O , Gat I , Bar-Kalifa E , Fitoussi P-Y , . Attachment-based family therapy for sexual and gender minority young adults and their nonaccepting parents. Fam Process 2022;61(2):530–48. 10.1111/famp.12770 35362553 PMC9325072

[29] Ford CL , Griffith DM , Bruce MA , Gilbert KL . Racism: science and tools for the public health professional. American Public Health Association; 2019.

[30] Hassen N , Lofters A , Michael S , Mall A , Pinto AD , Rackal J . Implementing anti-racism interventions in healthcare settings: a scoping review. Int J Environ Res Public Health 2021;18(6):2993. 10.3390/ijerph18062993 33803942 PMC8000324

[31] Huang YT , Ma YT , Craig SL , Wong DFK , Forth MW . How intersectional are mental health interventions for sexual minority people? A systematic review. LGBT Health 2020;7(5):220–36. 10.1089/lgbt.2019.0328 32412864

[32] Bazarsky D , Edwards BJ , Jensen L , Subbaraman S , Sugiyama B , Travers S . Standards of practice: core competencies for LGBTQIA+ directors and professionals in higher education. J Divers High Educ 2022;15(2):141–52. 10.1037/dhe0000282

[33] Byrd WC , Brunn-Bevel RJ , Ovink SM . Intersectionality and higher education: identity and inequality on college campuses. Rutgers University Press; 2019.

[34] National Association of Diversity Officers in Higher Education. A framework for advancing anti-racism strategy on campus. 2021. Accessed October 20, 2022. https://nadohe.memberclicks.net/assets/2021/Framework/National Association of Diversity Officers in Higher Education - Framework for Advancing Ant-Racism on Campus - first edition.pdf

[35] Nguyen TH , Samayoa AC , Gasman M , Mobley S Jr . Challenging respectability: student health directors providing services to lesbian and gay students at historically Black colleges and universities. Teach Coll Rec (1970) 2018;120(2):1–44. 10.1177/016146811812000201

[36] Jones B , Lo P , Wilkerson A , Xu A , Hall L , Cooper K , . Modeling inclusion: HBCUs and LGBTQ+ support. Accessed January 20, 2023. https://hrc-prod-requests.s3-us-west-2.amazonaws.com/CMSI-and-HRC-LGBTQ-Brief.pdf

